# Canadian farmers’ perceptions of social sustainability in agriculture

**DOI:** 10.1371/journal.pone.0299100

**Published:** 2024-04-26

**Authors:** Heather Heise, Felicia Hrvatin, Abbey Cran, June Matthews

**Affiliations:** School of Food and Nutritional Sciences, Brescia University College at Western University, London, Ontario, Canada; University of Wisconsin-River Falls, UNITED STATES

## Abstract

Sustainable food production is an important part of dietetic education and training; however, the focus in the dietetic sphere is often on the environmental aspect. Understanding the multi-dimensional nature of sustainability can enhance dietetic students’ sustainability competences–such as empathy and change of perspective, systems thinking, and critical thinking and analysis–to help them in their future careers and strengthen their position in society as trusted and knowledgeable food and nutrition professionals. Enhancing public understanding of sustainable food production is imperative as populations become more urban, are less connected to agriculture, and have expectations for sustainably grown/raised food, often without knowing current food production practices or the multiple aspects of sustainability that must be in place for farmers to meet those demands. The goal of this research was to understand Canadian farmers’ perceptions of environmental, economic, and social aspects of sustainable food production. Employing a descriptive qualitative approach and constant comparative analysis, four food and nutrition researchers analyzed interviews from 52 farmers from across Canada. Participants had to be English-speaking, produce food through farming on land, and own or rent the land on which they farm. Telephone/video interviews revealed five overarching social themes: (1) the importance of community and social capital, (2) public perception and social license to operate, (3) lack of infrastructure, and (4) deep connections to personal lives. The final theme, mental health issues (5), reflected the consequences of the multiple sources of stress that can undermine the social sustainability of farmers, farm communities, and food production. These findings may help various audiences appreciate the multiple dimensions of sustainable food production; reflect on their values, perceptions, and actions with regard to agriculture; and enhance their compassion and empathy for all farmers.

## Introduction

### Sustainable food production

Sustainable food production is vital for human survival; however, it means different things to different people. For example, while exploring consumers’ perceptions of words commonly associated with agriculture, several focus group participants indicated that they had never heard of the term ‘sustainable agriculture’ or were unsure what it meant [1]. Other participants who were familiar with the term explained it by saying: “Agriculture by its very definition is self-sustaining. You plant, you harvest, and you go back and plant and harvest, plant and harvest, you can’t be more sustainable” [1]. Many farms have also been in the same family for generations; thus, the term sustainable agriculture would benefit from additional information or context to provide clarity and shared understanding among producers and consumers [1]. Learning from farmers about their perspectives of sustainable food production would support this enhanced understanding.

From the seminal Brundtland Report in 1987 [2] to the Sustainable Development Goals adopted by all United Nations Member States in 2015 [3], there is broad agreement that there are three key aspects of sustainability: environmental, economic, and social. Each of these components is necessary for farmers, their communities, and overall food production to be sustainable; that is, able to meet the needs of the present without compromising the ability of future generations to meet their own needs [2]. Unfortunately, the aspect of sustainability most often emphasized is the environmental dimension [4]; however, transitioning toward more sustainable systems requires a clearer and more explicit recognition of the multi-dimensional nature of the concept of sustainability, particularly since the social dimensions of food systems receive insufficient attention [4].

The sustainability of Canadian agriculture is of interest to the 2 million Canadians who are involved in primary food production and related industries [5] and to the millions of people around the world who depend on the food produced by Canadian farmers. The Government of Canada recently implemented the Agri Communication Program to increase consumer awareness of environmental sustainability as it relates to agricultural best practices [6]; however, without helping the public also understand the social and economic pressures experienced by farmers, which often hinder the implementation of environmental strategies, non-farmers may never understand the challenges farmers face, nor how their action/inaction may undermine the sustainability of agriculture. Indeed, it will be difficult for farmers to achieve Sustainable Development Goal 3, Mental Health and Well-being [3] without also being in socially sustainable farms and communities; therefore, who better to elucidate this concept than farmers themselves?

### Stress associated with farming

It has long been documented that farmers experience many stressors and uncertainties (e.g., debt, weather, pests, markets, isolation, regulations) [7–10], most of which are inevitable and acceptable risks associated with farming. However, the persistent, frequent, and overwhelming nature of these pressures can result in stress responses (often referred to as ‘stress’) that consist of cognitive, behavioral, and biological changes [11]. Stress, in itself, is not an illness [12]; however, when additional chronic, variable, and unpredictable stressors (e.g., discrimination or bullying) are superimposed on day-to-day aggravations, failure to adapt results in distress and increased vulnerability to mental and physical illnesses [11]. Farmers have expressed feeling marginalized, undervalued, and misunderstood [13]. They have also experienced stigmatization at the individual, social, and institutional level, causing them to hide their profession in public, isolate themselves with other farmers among whom they feel more comfortable, or feel unhappy and worry about potential consequences of the stigmatization [14]. This does not bode well for the achievement of the Sustainable Development Goal of Mental Health and Well-being in the farming sector.

Finnish farmers have reported the “treatment of farmers in society and the media” as one of the most common sources of stress [15, p.263]. Several farmer witnesses at the Canadian House of Commons Standing Committee on Agriculture and Agri-food [16] acknowledged having the same experience. Several stated that activist groups use social media to put pressure on livestock producers. They described how they have been cyberbullied and that being targeted in this way has had a serious impact on their mental health. Indeed, research has shown that anxiety, depression, emotional exhaustion, and burnout are all higher among farmers than in other occupations or in the general population [17, 18]. Clearly, this has negative implications for the social sustainability of agriculture.

### Social aspects of sustainable food production

Social capital (e.g., personal networks, trust in people and institutions, reciprocity among individuals) provides support to help individuals overcome difficulties [19, 20]. Thus, social capital can promote the personal growth of individual farmers, regional economic development, and overall food production [21, 22]. Indeed, social capital is key to meeting social, economic, and environmental demands [23]. Social capital is a particularly important resource for small-scale farmers who have no hired labour; however, the increased size of farms and the decreased number of farmers means that owners of large-scale farms have fewer neighbors to draw on as well [24].

Janker and Mann [25] posit that a holistic concept of sustainability cannot neglect the social dimension. They analyzed how farm-related sustainability assessment tools defined and operationalized this dimension of sustainability. Four topics that were consistently associated with social sustainability in agriculture included human rights, work conditions, life quality, and impact on society. Social sustainability in agriculture, however, must also include society’s views of farming [26]. Farmers want the public to perceive them as credible and trustworthy, and to be confident that they are behaving in environmentally [27] and socially acceptable ways [28]. Gaining this approval represents social license, the implicit consent provided by the broader community to a business, project, or industry that allows its function [29].

A key component of social license to operate (SLO) is trust [29], but many factors can undermine this trust. For example, as each generation becomes further removed from food production, public knowledge about agriculture declines [30, 31]. In fact, 91% of Canadians know little or nothing about modern farming practices, a statistic that has not changed since 2016 [32]. A misinformed or underinformed public hinders the development of the trust that is necessary for social sustainability in food production. Although Canadian consumers rated farmers as the most trustworthy stakeholder in the food system [33], their trust has declined towards Canadian agriculture overall [33]. To build trust with farmers, agricultural sales representatives have been encouraged to understand the goals and missions of farmers’ operations [34]. The same can be said for enhancing public trust in agriculture: most people would benefit from increased understanding of agricultural production as well as farmers’ perceptions of the multiple aspects of sustainability that must be met for them to be able to produce food sustainably. To help to build trust between consumers and farmers, the agricultural industry can also provide evidence (e.g., third-party audits) to show that farmers are acting honestly and fairly [35].

It is also important to consider that, although social licence was “invented by Business, for Business” [36], it seems to be characterized mainly from a consumer perspective. Communities are demanding greater participation in decision-making and assurances that industry practices (particularly related to mining) are conducted safely and responsibly [37, 38]. However, as Prno [38] explains, “Only when a community feels *their vision* [our emphasis] of social, economic, and environmental sustainability is being supported, or at the very least isn’t being threatened, will they begin to contemplate issuance of a SLO” [p.586]. Farmers are well aware of SLO and work hard every day to earn it; however, as the public becomes more disconnected from food production, and more connected to social influencers who spread misinformation and disinformation, it is increasingly challenging for farmers to achieve social licence, regardless of how well they are implementing sustainable agricultural practices [27]. It would be helpful for the public to recognize the multiple aspects of sustainable food production, and that agriculture is not only an industry that extracts natural resources, but also one that preserves and enhances these resources, while at the same time producing food, feed, fuel, and fibre, as well as hundreds of byproducts from animal agriculture (e.g., medicines, detergents, personal hygiene products, sports equipment, adhesives, oils and lubricants, building materials, organic fertilizer).

### Dietitians’ role in sustainable food systems

Dietitians’ role in sustainability originated decades ago [39] and there is increasing interest in how dietitians can contribute to sustainable food systems. While some food and nutrition researchers encourage a comprehensive approach to understand and improve food systems [40–45], other food and nutrition researchers [46–51] and some dietetic association position papers [52, 53] focus on the environmental aspect. There are, however, social, economic, and environmental effects at every point in the system [42]. Even when the social aspect of sustainable food systems is included in dietetics standards (which form the basis for dietetic education and training), it focuses on the consumer perspective (e.g., “We shape policy decisions on diet and nutrition to promote a healthy and sustainable *society*” [their emphasis] [54]. Healthy and sustainable diets also differ in their availability, accessibility, and cost [55]. Furthermore, what is considered healthy is not always sustainable, and what is considered a sustainable diet is not always healthy [4]. There is also little/no mention of the social aspects that are necessary for farmers to be supported in achieving and maintaining sustainable food production–the foundation of all diets.

Dietitians work throughout the food system, which increases the need for them to have a broad understanding of sustainable food production. Although there are flaws in the present system, it delivers a constant supply of sustenance; therefore, those who propose changes must first understand the complexities and challenges of the existing system [42]. Thus, more training for dietitians in sustainable food production is recommended [40, 54, 56–60].

Students’ understanding of sustainable diets is often characterized by environmental aspects [61–65] or is limited to specific farming techniques (e.g., organic) [66]. Consumers’ perceptions of food-related sustainability are also related to environmental aspects [67, 68]. People trust health professionals to provide accurate, evidence-based, and trustworthy information; therefore, highlighting economic and social aspects of sustainable food production, in addition to the health and environmental aspects, is imperative [68].

Understanding the multi-dimensional nature of sustainability can enhance dietetics students’ sustainability competences [69]–such as empathy and change of perspective, systems thinking, and critical thinking and analysis–to help them value all stakeholders’ perspectives and position them as trusted and knowledgeable food and nutrition professionals. It has been proposed that all university students acquire critical food literacy training to empower them to incite change towards sustainable food production [70]; however, it is unclear if such a course would provide students with a thorough understanding of the multi-faceted dimensions of sustainability. Agriculture-related courses are already being taught in dozens of faculties beyond traditional departments of agriculture and science (e.g., sociology, philosophy, religion, gender studies) [71]. A content analysis of over 900 Canadian agriculture-related university course outlines in both science and social science faculties revealed that farmers’ mental health, a key component of the social sustainability aspect of food production, was rarely discussed [71]. Educating university students, as well as members of the public, about the multiple dimensions of sustainable food production is important as societies become more urban and people are less connected to agriculture. This could also enhance agriculture’s social licence to operate.

The goal of this research was to understand farmers’ perceptions of environmental, economic, and social aspects of food production. This paper focuses on the social aspect. The environmental aspect has been reported previously [27].

## Materials and methods

The researchers approached this study using a descriptive qualitative method [72]. This is a rigorous and useful research approach for graduate students to describe and summarize a phenomenon of interest [73]. Participants had to reside in Canada, be English-speaking, produce food through farming on land, and own or rent the land on which they farm. Recruitment occurred from January 29, 2019, to March 30, 2020, through a rural magazine advertisement and snowball sampling. A $25 honorarium was offered to each participant. The study, including the procedure for obtaining and documenting oral consent, was approved by the Non-Medical Research Ethics Board at Western University, Approval #112911.

The Letter of Information was read to each participant at the beginning of their online/telephone interview. The graduate student interviewers explained the risks and benefits of participating in the study; provided assurances that participants could withdraw at any time and/or not answer all questions; and described procedures for data security, confidentiality, and storage. Each participant then provided informed verbal consent, which was documented by the interviewers. Following a semi-structured interview guide (S1 File), the interviewers asked the following broad research questions:

- What comes to mind when I say the word sustainability?
- What does food production sustainability mean to you?
- How would you describe social sustainability as it relates to farming? Generic prompts (e.g., Can you tell me more about that? What do you mean by…?) encouraged participants to expand on their responses. The interviewers periodically summarized participants’ responses and asked for confirmation or clarification (i.e., member checking) to enhance trustworthiness and decrease researcher bias [74]. Participants were asked several demographic questions at the end of the interviews, the answers to which were also documented on hard copy.

All interviews (except one) were audio-recorded, transcribed verbatim by two graduate students and four undergraduate research assistants, independently coded line-by-line by the researchers, and analyzed concurrently throughout data collection using the constant comparative method [75].

Using Microsoft Excel to organize the data, initial themes were subsequently updated or expanded with sub-themes as additional data were collected and analyzed. A post-graduate student in the Diploma in Dietetic Education and Practical Training program was invited to independently conduct secondary analysis of the transcripts. Analyst triangulation [76] provided multiple ways to understand the data, minimized bias and selective perception, and contributed to a comprehensive analysis (i.e., credibility/internal validity) [75]. Trustworthiness was further enhanced in several ways. First, memo writing allowed the researchers to document emerging interpretations and decisions made throughout the research process (i.e., dependability/reliability) [77]. Second, in-depth descriptions of both the phenomenon (farmers’ perceptions of social sustainability) and the participants may help readers determine the applicability of the findings to their own context (i.e., transferability/external validity) [77]. Third, the student researchers and their faculty advisor met weekly during this iterative and reflective analytic process to de-brief and come to thematic consensus (i.e., confirmability / objectivity) [77]. Fourth, the student researchers discussed the research process and theme development amongst themselves during peer debriefing opportunities to enhance their interpretation of the data and the credibility of their research [78]. Finally, engaging in reflexivity allowed the student researchers and their faculty advisor to understand how their backgrounds and knowledge of the phenomenon (or lack thereof) influenced both process and outcomes [79]. Although the student researchers were not farmers, they were familiar with current issues in Canadian agriculture through participation in an undergraduate agriculture course. The faculty advisor is a Registered Dietitian and Professional Home Economist with a farming background and 20 years of experience in conducting qualitative research studies.

In this paper, themes and sub-themes are supported by representative quotations from one or more of the participants to add trustworthiness and transparency to the findings [80]. Including participants’ own voices deepens readers’ understanding of participants’ perspectives and experiences, reinforces the researchers’ interpretations, and shows the richness of the data [80]. To protect confidentiality, each participant was assigned a random number between 1 and 52, and representative quotations were labelled with a unique identifier (e.g., ON26F = Province, Participant #26, Female).

## Results

Forty-eight interviews were conducted with 52 farmers (four interviews had two participants each). The number of years that participants had been farming ranged from 2 to 50, representing, in total, over 1000 years of farming experience. Participant characteristics from the demographic questionnaire (S2 File) are presented in Table 1. Farm characteristics are presented in Table 2.

**Table 1:** Participant characteristics (n = 52).

Characteristic	% (n)
**Age (y)**	
18–29	15 (8)
30–39	25 (13)
40–49	23 (12)
50–59	17 (9)
60–69	13 (7)
70+	6 (3)
**Sex**	
Male	65 (34)
Female	35 (18)
**Education**	
High school	4 (2)
College/Apprenticeship	40 (21)
University	42 (22)
Graduate	14 (7)
**Marital Status**	
Married	75 (39)
Not married	25 (13)
**Off-farm work** ^**a**^	
Full-time	29 (15)
Part-time	27 (14)
None	44 (23)

^a^ Interviewees only, not spouses/partners

**Table 2:** Farm characteristics (n = 48).

Characteristic	% (n)
**Location**	
**Western Canada**	
British Columbia [BC]	8 (4)
**Prairie provinces**	
Alberta [AB]	8 (4)
Saskatchewan [SK]	7 (3)
Manitoba [MB]	13 (6)
**Central Canada**	
Ontario [ON]	54 (26)
**Eastern Canada**	
Nova Scotia [NS]	8 (4)
New Brunswick [NB]	2 (1)
**Size (number of acres owned and rented)**	
Small (<99 acres)	23 (11)
Medium (100–999 acres)	46 (22)
Large (≥1000 acres)	31 (15)
**Products**	
**Small**: primarily fruits and vegetables; some had animals	
**Medium**: fruits (apples, strawberries); vegetables (field-grown and greenhouse); grains, forages, and legumes; most had animals (diary, beef, laying hens, broiler chickens, sheep)	
**Large**: field crops (canola, wheat, corn, soybeans, barley, yellow peas, quinoa, flax, oats, mustard, and forages); a few had animals (laying hens, broiler chickens, and dairy/beef cattle)	

### Comparison to Canadian population

In comparison to the Canadian farm population, where 60 percent of farmers are 55 years of age or older [81], only 20% (10/52) of participants were older than 60 years of age. More participants (56%; 29/52) engaged in off-farm work than the Canadian farm population (47.7%), and a higher percentage had university degrees (56%; 29/52 vs. 18%) [82]. The percentage of participants who had college/apprenticeship (40%; 21/52) was comparable to the national farm population (35%) [82], as was the percentage of participants who identified as female farm operators (35%; 18/52 vs. 30.4%) [83].

### Themes

Five overarching and connected social themes were identified from participants’ responses: (1) the importance of community and social capital; (2) public perception and social license to operate; (3) lack of infrastructure; and (4) deep connections to personal lives. The final theme, mental health issues (5), summarized the consequences of the stressors and pressures that undermine the social sustainability of farmers and farm communities.

### 1. The importance of community and social capital

Participants talked about how they build social capital in their geographical and/or occupational communities: *“You gotta surround yourself by good people*, *and then they’ll help you put the systems in place to be sustainable”* (ON03M). Many spoke of their work with pride: *“[Being sustainable means] to make a living farming*, *do what is right for the land*, *and be able to pass on the farm to the next generation in good condition”* (NS52M). Another pointed out that farmers feel a responsibility that goes deeper than for many other occupations due to the vital importance of their industry: *“Okay*, *we need to make money like everybody else*, *but there’s a social responsibility to be in our position”* [ON03M]. Two sub-themes revealed that farmers value their local communities; however, smaller rural populations translate into a smaller voice.

#### Farmers value their local communities

Half of the participants (n = 24) said that they valued their communities and were proud of their level of civic engagement (e.g., donating food to food banks) to build *“the fabric of the community*” (ON04M). Buying local goods and services was important economically and socially, to expand the networks that create social capital: *“The non-farmers in the small town pretty much understand where I’m coming from*, *and I understand where they’re coming from*. *So*, *buy local*. *Support your local businessperson”* (AB34M).

#### Smaller populations equal smaller voice

Although many participants saw their communities as rich and fulfilling, twenty-four participants mentioned the declining farming population in Canada, which meant fewer volunteers to maintain community venues or step into leadership roles in agricultural organizations. This translated into broader negative consequences both socially, *“Farmers make up about 2% of the population*. *The other 98% makes the rules…and they don’t understand what we do”* (ON04M), and politically, *“Our vote doesn’t mean anything anymore”* (ON24M). Smaller populations also resulted in school closures, despite the valuable role they could play in educating youth about agriculture: *“As farmers*, *we always say they should be bussing kids outta the big schools into the rural community instead of the other way around”* (ON10F).

A smaller farming population also contributed to a decline in farmers’ voices in public spheres, and participants worried about the opportunity that creates for potentially misinformed opinions, particularly from celebrities: *“When Gwyneth Paltrow has some funny idea about things*, *she’s got a big platform that she can use*, *and my voice isn’t heard over someone like that”* (MB32M). Many talked about building public trust but stated it was *“getting tougher and tougher to tell our side”* (BC44F). Although some farmers were trying to expand their reach (e.g., through social media), not all participants saw this as particularly helpful. A grain farmer explained that they don’t have same influence as other industries:

*At the elevator*, *rail cars only come on the weekend*, *and they often come weeks late so our cash flow is controlled by the rail companies*. *That’s just not acceptable*. *But they have the lobbying power in Ottawa*. *We don’t*. (MB48M)

Adding to this problem was a pervasive feeling that the non-farming public does not understand agriculture and is increasingly withdrawing its acceptance of farm practices, the foundation of the second theme.

### 2. Public perception and social license

Over two-thirds of participants (n = 27) discussed public perception of farmers and whether non-farmers accepted or approved of them and their practices (i.e., social license to operate). One quote resonated throughout the interviews: “*A lot of people really don’t have any idea how things are produced”* (ON18M). Sub-themes included lack of public understanding and trust, disconnection between farmers and non-farmers, and policies that are not conducive to farmer well-being and/or sustainable agriculture.

#### Lack of public understanding and trust

Although they were already engaging in a variety of sustainable farming practices, or learning new ways to make agriculture more sustainable, participants perceived their work was threatened by a lack of public understanding:

*The biggest issue is not to make our current food system sustainable*. *It’s to get people to understand that*, *by and large*, *it IS sustainable*. *I don’t understand why farmers are being vilified for our use of synthetic fertilizers*, *pesticides*, *and antibiotics*, *and hormones*, *and all the things they’ve got us nailed down for*. *Those things all got there because they are*, *in fact*, *best practices* (ON04M).

Participants mentioned a variety of sustainable farming practices related to soil, water, energy, and biodiversity. Many felt confused and frustrated: *“We feel a lot of pressure from the public to be more sustainable*, *but the things we do don’t get recognized when we do them”* (ON01M), partly because *“a lot of aspects of farming are hidden from the public just through lack of knowledge transfer”* (BC43M). Public criticism of food grown in greenhouses was troubling to greenhouse operators: *“They don’t understand that greenhouses produce ten times the yield per square metre”* (ON31M).

Participants also lamented the declining level of public trust–the key component of social license to operate–in farmers’ expertise to produce safe and abundant food. As one farmer explained,

*Farmers used to have some of the highest trust levels of any industry*. *And it’s just gone down and down and down*. *Because there’s less trust*, *some people think we’re destroying the world and we’re all evil*. *The public wants to change things without really talking to us ‘cause they don’t trust us anymore and they think we’re doing everything wrong* (ON07M).

Another said, *“Farmers don’t understand where they became a four-letter word”* [ON09M], which, for some, resulted in feelings of regret: “*When I started farming*, *if I knew that people would have such a great mistrust of my profession*, *I would have strongly reconsidered becoming a farmer”* (BC43M).

To gain social license, some farmers encouraged their peers to communicate with non-farmers at every opportunity:

*Social sustainability means that what I do has to be acceptable to people who don’t do what I do*. *But that means I have to put a bit of effort in getting them to understand why we do what we do*, *how we do it*, *and some transparency so they can understand it* (MB32M).

Educating the public and maintaining their trust may be necessary for farmers to continue doing their jobs in a sustainable and efficient way. The extra work required, however, can be taxing: *“Farmers are doing so much work already*, *and they’re usually exhausted*, *so that adds a new layer to the job”* (BC43M). A farmer from the opposite end of the country agreed that people should ask their local farmers if they have any questions; however, she feels *“a lot of pressure”* to speak to school groups or give farm tours, and “*it can be hard to do all of it”* (NS27F).

#### Disconnection between farmers and non-farmers

Underlying farmers’ perceptions that they are given limited social license to operate was a sense of disconnection between farmers and non-farmers. One farmer repeatedly expressed the need to prove that his practices are sustainable:

*To be socially sustainable means to prove that what we’re doing is environmentally sustainable*. *That’s what a lot of people are concerned about so being able to prove that I do care about my hens and that I do care about my soil*. *So*, *educate and prove to people that I am doing something that they want me to do*, *that they can trust what I’m doing* (MB48M).

Another spoke confidently about his operation: “*I know I do a great job*. *I’m camera-ready all the time*.*”* (ON24M)

Increasing migration from urban to rural communities presented additional challenges. A beef farmer stated that to be socially sustainable within her community, some effort on behalf of non-farmers was needed: “*People shouldn’t complain about us*. *If you live in an agricultural community*, *then you need to accept that [noise and odor] happens”* (AB14F).

One farmer likened public perception and social sustainability to a marriage, where both sides must work at it to be successful. Another acknowledged that dialogue is needed:

*You have that passionate farmer on one side of the line*. *And then you have that passionate person way on the other side of the spectrum*. *The closer we get to the line [in the middle]*, *the more socially sustainable we get*. *There’s always that bad person*, *right*? *On both sides of the line* (ON09M).

#### Policies that are not conducive to farmers/agriculture

Several participants noted that the public’s lack of trust in the professional knowledge of farmers can result in policies that are not conducive to farmers or agriculture. For example, banning glyphosate was seen as a backward step that would result in farmers reverting to less sustainable practices in all three spheres (economic, environmental, social): *“They [the public] don’t understand how glyphosate’s used and how it is in the environment and say*, *‘We don’t want it’*. *So*, *we lose our biggest [crop protection] tool and that causes a lot of stress”* (SK36M).

Government officials who did not understand agriculture were another challenge: *“Sometimes it feels like the people with the least education are deciding what we are allowed to do”* (MB48M). Examples included policies that dictated an arbitrary calendar date (not weather, wind, or soil conditions) when manure, an organic fertilizer, could be applied to their land; policies that prevented them from draining some fields to increase production and prevent farm equipment from getting stuck; or policies that prohibited them from drilling a well. This lack of engaged governance, coupled with a small farming population, reflected another troubling reality for many participants: lack of infrastructure, the essence of the third theme.

### 3. Lack of infrastructure

This theme came up in half of the interviews (n = 26). One participant explained that although farmers pay property taxes, they do not have the utilities (e.g., natural gas, municipal water) that urban dwellers take for granted. Lack of high-speed, or even reliable, internet and cell service beleaguered many of them. Cell phones were considered essential for farmer and animal safety and well-being (e.g., using Snapchat to send pictures from a back field to confirm if lambing was proceeding normally). Many participants (n = 18) used practices that required reliable access to these services. A farmer in Eastern Canada said, *“There is no high-speed internet here*. *We are on Broadband*. *The [national regulatory authority] feels that you are underserved if you have less than 5 MG of download speed*. *We might have 1*.*5”* (NS46F). Farmers in Western and Central Canada experienced the same problems: “*My son says he’s had free internet in the middle of a stone castle in Europe that’s faster than what he’s paying 80 bucks a month for here [laughs]”* (MB32M) Another said, *“My Wi-Fi’s probably slower than what you’d get at a [coffee shop] for free*. *I sent my tech guy a screen shot of my upload/download speed and he’s like*, *‘How do you work in a day*?*’* (ON26F).

Any drawbacks to living in a rural area were outweighed by the benefits of not living in a city; however, this was not considered an excuse for governments to abdicate their responsibility to provide essential services. In addition to feeling marginalized in terms of service provision, some participants expressed the challenges that come with having a business so closely connected to their personal lives and discussed violations of their right to live free from harassment, both of which constituted the basis for the fourth theme.

### 4. Deep connections to personal lives

Participants identified many challenges with having work and personal lives so closely intertwined. These fell into three sub-themes: family tensions and the overlap between work and family life, the importance of economic sustainability, and personal harassment based on their occupation.

#### Family tensions and overlap between work and family life

Most farms are family businesses, which can result in tensions with little opportunity to decompress or recoup: *“What people forget is that farmers live where they work*. *It’s the 24/7 and that impacts the whole family*. *How do you not talk about these things*? *There’s nowhere to escape”* (ON20F).

Planning for the next generation to assume ownership of the farm can also be fraught with tension. The dilemma created when their son expressed interest in taking over the family farm was voiced by one farmer in this way: *“How can our daughter*, *who doesn’t want the farm*, *feel valued in this process*?*”* (AB16F). Participants spoke of some parents who wanted to control the farm longer than was prudent, or who perceived that the next generation lacked expertise or drive, which can be socially devastating: *“More families have been ruined [during succession planning] than businesses”* (ON26F). For those who did continue, family expectations created an additional burden, as no farmer wanted to be the one who ‘lost the family farm’: “*Farms are often in the family for three or four generations*, *and there’s this stigma that you have to continue*. *And that can put a lot of stress and strain on top of the financial strain”* (ON26F).

#### The importance of economic sustainability

Underpinning most interviews were comments relating to economic stability. Worry about this aspect of farm life puts *“tremendous mental pressure on a farmer and their family”* (ON25M), as farming was perceived as *“one of the highest stress jobs for the least amount of economic returns”* (ON10F). One young farmer stated: *“You start grinding your teeth every night when it doesn’t rain for three weeks and realize maybe you shouldn’t be too [financially] leveraged”* (SK33M). Farmers also felt discouraged with how they were compensated for their work: “*We get 5 cents of a loaf of bread*. *It makes you sick as a farmer to know that”* (AB40M). Another explained:

*People don’t understand that you work all year*. *You put all the money out*, *but you only get paid if that crop comes off*. *It’s like you working all year and*, *if you don’t perform well for that last couple [of] days*, *you don’t get any salary at all* (ON23M).

Full-time off-farm work was a reality for more than half of the participants (n = 29), which contributed to stress. For one young farmer, economic insecurity forced many of her farm friends to abandon farming, *“and that definitely wasn’t by choice”* (BC44F). Participants acknowledged the effect of long hours and hard work, just to make ends meet: *“It’s a lot more than most people could ever handle and it’s unsustainable on our bodies”* (BC41F).

#### Personal harassment based on their occupation

Some participants, particularly farmers who raised livestock, spoke of personal harassment based on their occupation, and its impacts:

*[I know] a really*, *really*, *beautiful family that puts a lot of emphasis on how humane their animals are treated*. *They’ve had death threats sent to their door*. *When you’re farming in that kind of an environment*, *it takes a very big toll on your mental health* (BC44F).

She continued with a plea for more tolerance and understanding:

*We’re constantly vilifying people’s choices like*, *‘You need to be vegan because it’s better for the planet’*. *Farmers have a very difficult job*, *and I don’t think it’s going to get any easier in the future*. *Our population isn’t getting smaller*, *and we do have to be able to feed everybody*. *I don’t think that people who are choosing a plant-based diet are looking at things the wrong way and I don’t think that people who choose to consume animal products are doing the wrong thing either*. *Maybe stop being so judgemental and try to be nicer to everybody because we’re all just trying to do the best that we can* (BC44F).

Another farmer, while he was spraying a legal product on a calm and clear day, expressed frustration after being harassed by a neighbor:

*He’s telling ME he’s worried about the one in a million [risk] in the spray*, *but he’s holding a baby in one arm*, *and a cigarette in the other*! *I try to remain open-minded*, *and I always defer to science*, *but how do you argue with that*? *It’s not based on reason* (ON23M).

### 5. Mental health issues

The ambiguity, uncertainty, and uncontrollability surrounding anticipated stressors such as trespassers onto their farms or attacks on social media also contributed to stress responses (e.g., worry, loss of sleep). Lack of financial stability prevented community involvement or the pursuit of *“other relationships*, *other than just the farm”* (BC41F) to alleviate loneliness. Farm location also contributed to feelings of isolation: *“We can’t see anybody from where we live*. *So*, *yah*, *it can get a little lonely”* (ON18M). Deeply troubling were comments related to a failure to adapt to stressors, resulting in distress and associated negative mental health outcomes–the core of this last theme.

Over one-third of participants (n = 15) mentioned mental health and/or suicide. The importance of this dimension of social sustainability can be seen in one farmer’s definition of social sustainability:

*If you have a farm where you can make a living and a community where you’ve got neighbors who can back you*, *and you’ve got a place where you can be proud of what you do*, *and people are somewhat respectful of what you do*. *That’s what gives us mental health or takes it away from us* (ON10F).

Heavy workload combined with inadequate compensation, as well as the pressures associated with risk of catastrophic losses, were responsible for much of their distress: “*You can work so hard and seemingly do everything right and still barely scrape by*. *I know a number of people that have committed suicide because of the stress”* (ON18M).

One participant pointed out that efforts are being made to lower the rates of farmer suicides: *“Some of the Ag communities are now trying to do some training workshops for farmers to help them recognize signs in their neighbors before the next gun goes off”* (ON10F). Unfortunately, even if there are mental health supports, the lack of privacy in small towns can prevent farmers from seeking assistance: *“We’ve had suicides*, *you know*, *but it’s hard to reach out for help”* (ON12M). Furthermore, the care may not be appropriate for the farming community: *“What’s their expertise from an agricultural perspective*? *‘Cause the pressure and all of that is a bit different [for farmers] than other challenges a lot of people would have”* (ON25M).

In summary, many external stressors and pressures are compromising the social sustainability of Canadian farmers, farm communities, and overall food production. The ways in which these findings relate to the literature and to dietetic education and practice will be discussed next.

## Discussion

These findings complement previous research, contribute to our understanding of sustainable food production, and support the recommendation that definitions of, and recommendations for, sustainable food production be made in collaboration with food producers [42].

Participants’ comments confirmed the dimensions of loneliness described in Wheeler et al.’s [84] study of British farmers. For example, smaller farm communities, a sense of disconnection, lack of public understanding, as well as the policy pressures described by farmers in this study reflected the same characteristics of cultural loneliness in Wheeler et al.’s research [84]. Cultural loneliness could be lessened by increased levels of public trust in the work that farmers do. Dietitians can help with this by communicating evidence-based information and being informed by people who engage in actual food production. The recent COVID-19 pandemic highlighted the indispensable nature of farmers’ work and prompted consumers to appreciate “…the fragile nature of global supply chains that were previously taken for granted” [85, p.111]. Thus, greater public respect for the agriculture sector may lessen these feelings of cultural loneliness. Participants’ descriptions of business-related stress, the blurred boundaries between home and work, and family tensions/expectations were representative of Wheeler et al.’s emotional dimension of loneliness [84]. Similarly, the long working hours, lone working, and geographic isolation described by these Canadians farmers also reflected British farmers’ social loneliness [84]. Enhanced empathy, developed through education on all aspects of sustainable food production, could also support dietary recommendations that value the context of food production and consider farmers’ perspectives and realities.

Echoing livestock farmers in Australia, participants in this study also expressed concerns about activists’ use of social media to communicate misinformation about livestock production [28]. One participant’s comment that he is “camera-ready all the time” appears to be a defensive response to misinformation and disinformation about farming practices, and the misperception that biosecurity protocols are hiding bad practices. Public education about biosecurity and the many third-party audits performed on all types of farms may lessen this constant need for farmers to prove that what they are doing supports animal health and welfare. Increasing consumer knowledge about production methods, however, may not result in the attitudinal changes that producers desire and may foster more polarized attitudes, particularly as community values shift (e.g., the move to veganism/vegetarianism) [28]. Perhaps focusing on the social aspect of sustainable food production could facilitate the identification of shared values between producers and consumers, increase community trust, and support farmers’ social licence to operate.

Frustration with policies not conducive to agriculture has been reported in other studies as well. For example, food donation tax credits for farmers to address food loss/waste and food insecurity (topics commonly covered in dietetic education and training) failed to solve the root causes of these problems [86]. Policy makers did not acknowledge that farmers were already donating food and that food banks could not handle large volumes of perishable produce. Furthermore, the 25% tax credit for donated food was insulting: it suggested to farmers that their work/produce was not appreciated enough to be credited at 100% of its value. Dietitians, therefore, can take a systems perspective by advocating for farmers’ meaningful participation in policy making and appropriate compensation for their time and expert knowledge. Dietetic educators can support students’ critical thinking skills through class discussions around the appropriateness of using on-farm food loss/waste as a solution to food insecurity.

Sensitivity training for different groups and cultures is included in many health professional programs, but these same professionals are not taught that agriculture is a unique culture [87]. Thus, to contribute to dietitians’ understanding of food production, it may be helpful to educate and train dietetic students on rural cultural competence [88]. Appreciating the complexity of food production and developing empathy for some of the pressures that farmers face might bestow “farm credibility” [89, p.119] on these health professionals. This, in turn, might enhance social capital [19, 20] in farm communities, whereby farmers could trust that dietitians are delivering not only evidence-based recommendations about food and nutrition, but also some information about the social and economic aspects that are necessary for food production to be sustainable.

Dietetic competencies [45] now include references to sustainable food systems, and dietitians are expected to “consider both nutritional and environmental science in order to give advice about sustainable diets” [41, p.10]; however, the social and economic sustainability of the people who produce food should be included in dietetic education and training as well. Dietetic educators can enhance their curriculum by including farmers’ voices. Dietetic students can engage in farm tours, advocate for mandatory rural placements during practicum training, and challenge prevailing narratives that validate only certain types of agricultural practices (e.g., organic) as sustainable. This type of engagement may enhance dietitians’ understanding of food production and the challenges farmers face and could potentially alleviate some of the burden that farmers carry in conveying information and fighting misinformation around farming practices. It may also encourage new graduates to pursue employment in rural, remote, and northern areas, where the shortage of healthcare professionals is particularly severe [90]. Finally, it may promote shared values [28]–areas of commonality between farmers and non-farmers–that will enrich and advance conversations about a common goal: sustainable food production.

In conclusion, sharing farmers’ perspectives can lend an authentic dimension to dietetic education and potentially increase students’ and the public’s appreciation for all aspects of sustainable food production. This is particularly important given the influence that public opinion has on policy making [91]. These results also highlight the need for dietitians to continue providing evidence-based practice so that their patients/clients/followers can make food decisions based on facts. Future research can examine how dietitians represent agriculture on their social media accounts and investigate how their education supports their understanding of food production.

### Strengths and limitations

Participants represented a variety of farmers in terms of age, sex, and type of farm, as well as different geographical regions of Canada. Diligent recruitment also resulted in a respectable sample size for a qualitative research project. Analyst triangulation [76] contributed to a comprehensive analysis [77]. More experienced interviewers may have gathered more detailed data. Self-selection bias was a concern as only farmers who were willing to be interviewed participated in the study. Most farmers were white and lived in Ontario. Unfortunately, no farmers in three territories or two provinces were recruited. A larger and more diverse sample would allow analysis by sub-populations (e.g., demographics, farm structure). It is worth noting that, despite these limitations, clear trends across regions were apparent.

## Supporting information

S1 File Semi-structured interview guide. (DOCX)

S2 File Demographic questionnaire. (DOCX)
